# Supplementary material for: Association between oral microbiome and seven types of cancers in East Asian population: a two-sample Mendelian randomization analysis
Source: Front Mol Biosci. 2023 Nov 21;10:1327893. doi: 10.3389/fmolb.2023.1327893 (PMC10702768; doi:10.3389/fmolb.2023.1327893)
Supplement: Supplementary file 1 [file DataSheet1.ZIP › Supplementary Materials/MR plots of saliva/saliva═╝/Pancreatic cancer/pheno.2183_to_pancreatic cancer_forest.pdf]

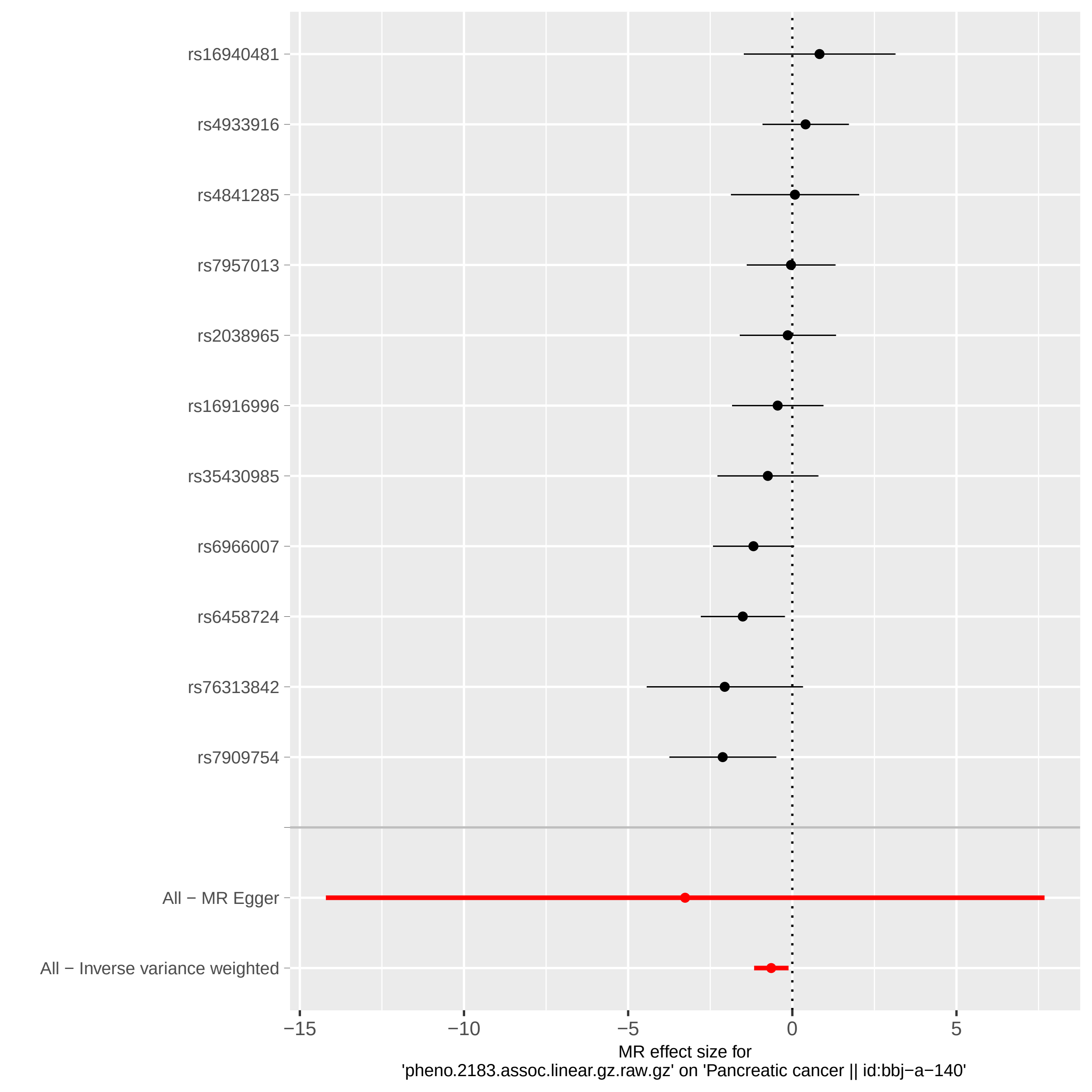
rs16940481
rs4933916
rs4841285
rs7957013
rs2038965
rs16916996
rs35430985
rs6966007
rs6458724
rs76313842
rs7909754
All − MR Egger
All − Inverse variance weighted
−15
−10
−5
0
5
MR effect size for
'pheno.2183.assoc.linear.gz.raw.gz' on 'Pancreatic cancer || id:bbj−a−140'
